# Effects of preoperative nutritional support combined with esketamine on recovery and analgesia after thoracoscopic radical resection of lung cancer in elderly patients

**DOI:** 10.3389/fsurg.2025.1684166

**Published:** 2026-01-07

**Authors:** Yu Hou, Zhiru Zhan

**Affiliations:** 1 Department of Anaesthesia, The First Hospital of Shanxi Medical University, Taiyuan, Shanxi, China; 2 Department of Operating Room, Zhongshan Hospital Affiliated to Xiamen University, Xiamen, Fujian, China

**Keywords:** lung cancer, thoracoscopic radical resection, preoperative nutritional support, esketamine, analgesia, immune function

## Abstract

**Aim:**

This study aims to explore the effects of preoperative nutritional support combined with esketamine on recovery and postoperative pain management in elderly patients undergoing thoracoscopic radical resection for lung cancer.

**Methods:**

A total of 165 elderly patients with lung cancer who underwent thoracoscopic radical resection at our hospital between June 2021 and March 2023 were enrolled and divided into a study group (SG, *n* = 85) and a control group (CG, *n* = 80). The SG received patient-controlled analgesia with esketamine, sufentanil, dexamethasone, and dexmedetomidine, while the CG received sufentanil, dexamethasone, and dexmedetomidine. The SG received nutritional support both pre- and postoperatively, whereas the CG received it only postoperatively. We compared resting and coughing visual analog scale (VAS) scores; Ramsay sedation scores at 6, 12, 24, and 48 h post-surgery; the number of analgesic pump compressions and drug consumption within 48 h; adverse reactions; recovery indicators; immune function; nutritional status; and quality of life between the groups.

**Results:**

Compared with the CG, the SG demonstrated significantly lower VAS scores at rest and during cough and higher Ramsay sedation scores at 6, 12, 24, and 48 h postoperatively (*P* < 0.05). The SG also required fewer analgesic pump compressions and lower analgesic drug dosages within 48 h (*P* < 0.05). The incidence of adverse reactions within 48 h was lower in the SG (*P* < 0.05). Postoperative recovery indicators, including time to first exhaust, first defecation, and hospitalization, were significantly shorter in the SG (*P* < 0.05). Furthermore, the SG showed significantly elevated levels of Immunoglobulin G (IgG), IgM, IgA, albumin (Alb), transferrin (TRF), and prealbumin (PAB) (*P* < 0.05), as well as higher scores across all 36-Item Short Form Health Survey (SF-36) dimensions (*P* < 0.05).

**Conclusion:**

Preoperative nutritional support combined with esketamine was associated with reduced pain, improved postoperative recovery indicators, better maintained immune function and nutritional status, and a higher quality of life in elderly patients after thoracoscopic radical resection of lung cancer.

## Introduction

Lung cancer is a leading cause of cancer-related mortality worldwide, particularly among the elderly population (1). Radical resection is the primary treatment for stage I and II lung cancer (2), with reported 5-year survival rates reaching up to 80% and generally favorable prognoses (3). Thoracoscopic radical resection has become a common surgical approach offering advantages over traditional thoracotomy, such as less trauma and shorter operation duration (4). However, elderly patients often present with compromised physiological reserves and comorbidities such as chronic obstructive pulmonary disease (5), making them highly vulnerable to postoperative complications arising from surgical stress and pain, such as respiratory dysfunction and delayed recovery, which prolong hospitalization and increase burdens (6).

Effective postoperative analgesia is crucial yet challenging. While opioids are potent analgesics, their use, especially in the elderly, is limited by dose-dependent adverse effects, including respiratory depression, nausea, and the risk of tolerance (7, 8). This underscores the importance of multimodal analgesia strategies. The inclusion of N-methyl-D-aspartic acid (NMDA) receptor antagonists such as esketamine is recommended to improve analgesic efficacy and reduce opioid consumption (9). Esketamine, a novel NMDA receptor antagonist, provides effective analgesia with a favorable safety profile, potentially benefiting elderly patients (10).

Parallel to pain management, nutritional status is a key determinant of surgical outcomes. Surgical stress can lead to a catabolic state and malnutrition, impairing immune function and tissue repair, which is particularly detrimental in elderly cancer patients (11). Although enteral nutrition is essential, postoperative administration alone is often hampered by gastrointestinal intolerance (12). Evidence suggests that actively correcting the nutritional status before surgery and allowing the intestine to adapt to the enteral nutrient solution in advance can improve the compliance of postoperative enteral nutrition (13).

Therefore, we hypothesized that combining preoperative nutritional support with an esketamine-based analgesic regimen would synergistically improve recovery in elderly patients undergoing thoracoscopic lung cancer surgery. This study aimed to explore the effects of this combined approach on analgesia, recovery, immune function, and quality of life.

## Methods

### Study patients

A total of 165 elderly patients with lung cancer who underwent thoracoscopic radical resection at our hospital between June 2021 and March 2023 were enrolled. Inclusion criteria were as follows: (1) age >60 years; (2) pathological confirmation of lung cancer and scheduled for thoracoscopic radical resection; (3) complete clinical data; and (4) tumor stage I or II. Exclusion criteria were as follows: (1) history of radiotherapy or chemotherapy; (2) other concurrent cancers; (3) chronic pain history, long-term analgesic use or alcohol abuse; (4) allergy to study medications; (5) psychiatric disorders; or (6) severe systemic diseases. Patients were divided into a study group (SG, *n* = 85) and a control group (CG, *n* = 80) based on the analgesia protocol. The SG comprised 45 males and 40 females, with a mean age of 68.46 ± 6.35 years (range: 61–82) and 46 stage I/39 stage II cases. The CG comprised 42 males and 38 females, with a mean age of 68.53 ± 6.42 years (range: 62–80) and 45 stage I/35 stage II cases. No significant differences in baseline characteristics were observed between groups (*P* > 0.05), indicating comparability. All participants provided written informed consent.

### Anesthesia methods

All patients fasted and abstained from fluids before surgery. Blood pressure (BP), electrocardiogram (ECG), and blood oxygen saturation (SpO_2_) were monitored.

Peripheral venous access was established. Under local anesthesia, all patients underwent radial artery puncture for invasive arterial pressure monitoring. Combined inhalational and intravenous anesthesia was administered. Anesthesia was induced with intravenous sufentanil (0.4–0.5 µg/kg), etomidate (0.15–0.30 mg/kg), and rocuronium (0.6–0.9 mg/kg). A double-lumen bronchial tube was inserted, with placement confirmed by fiber-optic bronchoscopy before initiating mechanical ventilation. Anesthesia was maintained using intravenous infusions of propofol [4–8 mg/(kg·h)], remifentanil [6–10 µg/(kg·h)], dexmedetomidine [0.25–0.5 µg/(kg·h)], and cisatracurium [0.1 mg/(kg·h)], supplemented with 1%–2% sevoflurane inhalation. Intraoperatively, BP and heart rate were maintained within ±20% of baseline, and nasal temperature was kept at 36°C−37°C by adjusting fluid administration and using vasoactive drugs as needed. Sufentanil 10 µg was injected intravenously before the end of the operation. After patients woke up, the tracheal catheter was removed, and they were sent to the recovery room.

### Analgesia methods

Postoperative analgesia was administered via a patient-controlled intravenous analgesia (PCIA) pump. CG: sufentanil (Yichang Renfu Pharmaceutical Co., Ltd., specification 50 µg: 1 mL) 1.5 µg/kg + dexamethasone 5 mg + dexmedetomidine 100 µg.

SG: esketamine (Jiangsu Hengrui Pharmaceutical Co., Ltd., specification 50 mg: 2 mL) 0.2 mg/kg + sufentanil 1.3 µg/kg + dexamethasone 5 mg + dexmedetomidine 100 µg.

### Preoperative nutritional support methods

The SG received enteral nutritional support both pre- and postoperatively. One week before surgery, patients received 1,000 kcal/day of Weikaneng Balanced Nutrient (Harbin Byronst Clinical Nutrition Co., Ltd.; 1.0 kcal/mL) orally, in addition to a liquid diet. Postoperatively, on day 1, patients received 250 mL of isotonic sodium chloride solution. On day 2, they received 500 mL of Weikaneng Short Peptide (Harbin BST Clinical Nutrition Co., Ltd.; 0.85 kcal/mL) administered via nasogastric tube using an enteral nutrition pump over 24 h, along with 250 mL of isotonic sodium chloride solution. On day 3, the regimen consisted of 500 mL Weikaneng Short Peptide plus 500 mL Weikaneng Balanced Nutrient. From day 4 to day 9, patients received continuous 24 h infusion of Weikaneng Balanced Nutrient.

The CG received postoperative enteral nutritional support only, following the same postoperative protocol as the SG from day 1 onward.

The nutritional support regimen was administered as a fixed-dose protocol according to our institutional clinical pathway for perioperative care and was not adjusted based on individual patient body weight. Adherence to the oral and enteral nutrition protocol was monitored and recorded by the ward nursing staff. Compliance was defined as consumption of >90% of the prescribed volume.

### Observation indicators

(1) Pain and sedation: Visual analog scale (VAS) scores (at rest and during cough) and Ramsay sedation scores were recorded at 6, 12, 24, and 48 h postoperatively.
(2) Analgesic consumption: The number of analgesic pump compressions and total analgesic drug dosage within 48 h after surgery were recorded.
(3) Adverse reactions: The incidence of adverse reactions within 48 h, including respiratory depression, hypotension, nausea and vomiting, and dizziness, was recorded.
(4) Recovery indicators: Postoperative recovery was assessed by measuring the time to first exhaust, first defecation, and the duration of postoperative hospitalization.
(5) Immune function: Serum levels of immunoglobulin M (IgM), G (IgG), and A (IgA) were measured using immunoturbidimetry.
(6) Nutritional status: A 5 mL sample of cubital venous blood was collected. Nutritional markers—albumin (Alb), transferrin (TRF), and prealbumin (PAB)—were analyzed using an AU5400 automated biochemical analyzer.
(7) Quality of life: Quality of life was assessed using the 36-Item Short Form Health Survey (SF-36) prior to discharge. The SF-36 evaluates eight domains: physical functioning, role—physical, bodily pain, general health, vitality, social functioning, role—emotional, and mental health.

### Statistical analysis

Data were analyzed using SPSS software (version 24.0). Continuous data were presented as mean ± standard deviation (SD) and were compared using the *t*-test. Categorical data were expressed as numbers and percentages (*n*, %) and were compared using the chi-squared (*χ*^2^) test. A *P*-value of <0.05 was considered statistically significant. All analyzed variables had complete data for the 165 included patients. Therefore, no specific methods for handling missing data were employed.

## Results

### VAS scores and Ramsay sedation scores in both groups

**Figure 1:**
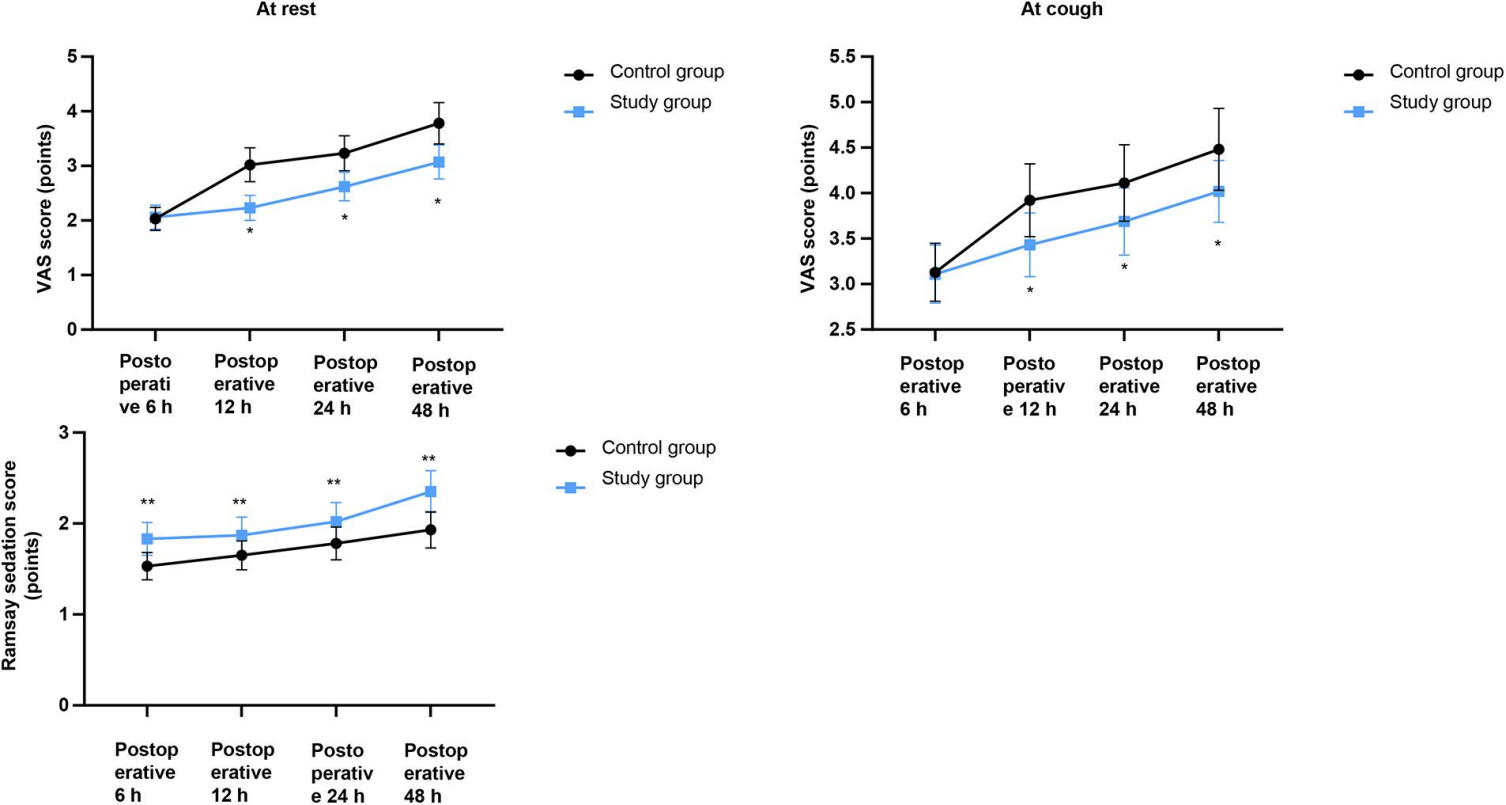
VAS scores and Ramsay sedation scores in both groups. VAS, visual analog scale. Data were presented as mean ± standard deviation. SG, study group [esketamine-containing analgesia (esketamine + sufentanil + dexamethasone + dexmedetomidine) combined with preoperative and postoperative enteral nutrition support]; CG, control group [standard analgesia (sufentanil + dexamethasone + dexmedetomidine) combined with postoperative only enteral nutrition support]. *P < 0.05.

As shown in Figure 1, the SG had significantly lower VAS scores at rest and during cough at 6, 12, 24, and 48 h postoperatively compared with the CG (*P* < 0.05). Concurrently, the SG also demonstrated significantly higher Ramsay sedation scores at all corresponding time points (*P* < 0.05).

### Number of analgesic pump compressions and the dosage of analgesic drugs within 48 h after surgery in both groups

**Figure 2:**
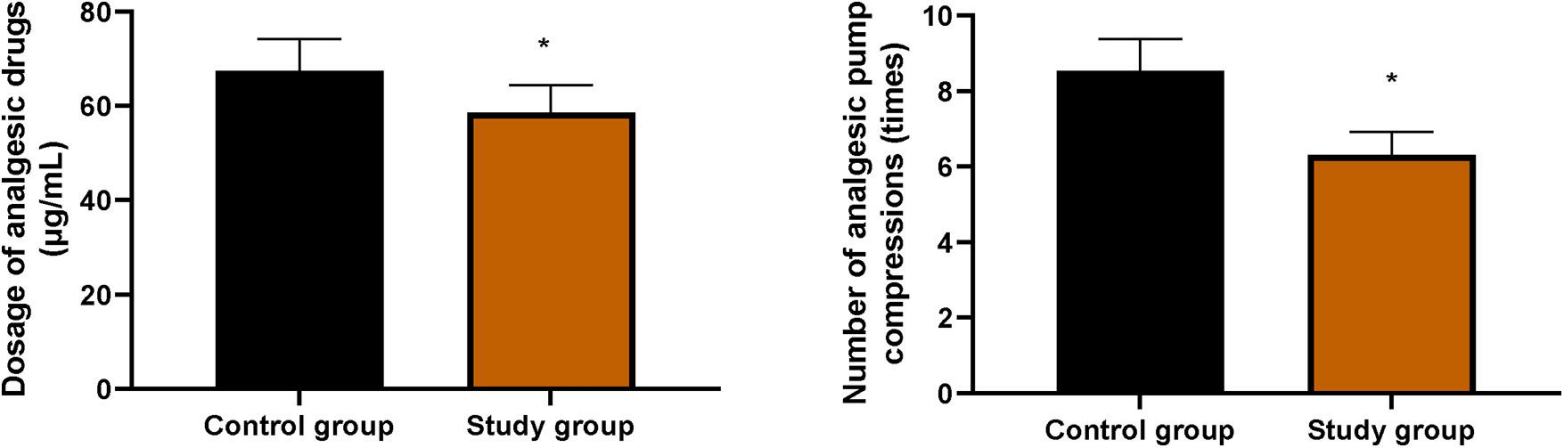
Number of analgesic pump compressions and the dosage of analgesic drugs within 48 h after surgery in both groups. Data were presented as mean ± standard deviation. SG, study group [esketamine-containing analgesia (esketamine + sufentanil + dexamethasone + dexmedetomidine) combined with preoperative and postoperative enteral nutrition support]; CG, control group [standard analgesia (sufentanil + dexamethasone + dexmedetomidine) combined with postoperative only enteral nutrition support]. *P < 0.05.

Figure 2 shows that the SG required significantly fewer analgesic pump compressions and a lower total analgesic dosage within 48 h postoperatively compared with the CG (*P* < 0.05).

### Occurrence of adverse reactions within 48 h after surgery in both groups

Table 1 demonstrates a significantly lower incidence of adverse reactions within 48 h postoperatively in the SG compared with that in the CG (*P* < 0.05).

**Table 1:** Occurrence of adverse reactions within 48 h after surgery in both groups.

Groups	Cases	Respiratory depression	Hypotension	Nausea and vomiting	Dizziness	Total incidence rate
Control group	80	3	2	2	2	9 (11.25%)
Study group	85	1	0	1	1	2 (2.35%)
*χ* ^2^						5.24
*P*						0.02

*P* < 0.05 compared with the control group (*χ*^2^ test).

### Postoperative recovery indicators in both groups

**Figure 3:**
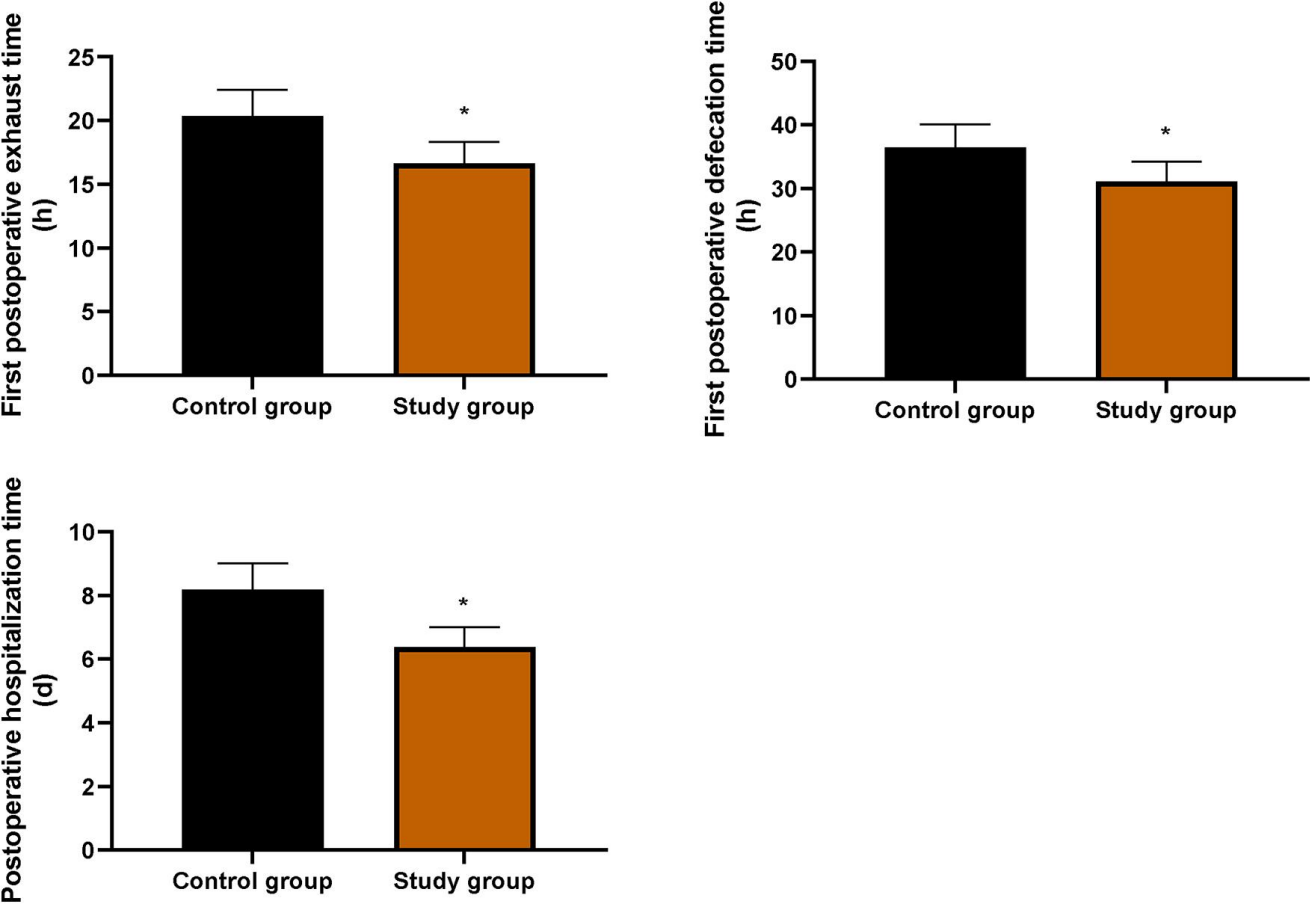
Postoperative recovery indicators in both groups. Comparison of the first postoperative exhaust time, first postoperative defecation time, and postoperative hospitalization time between the two groups. Data were presented as mean ± standard deviation. SG, study group [esketamine-containing analgesia (esketamine + sufentanil + dexamethasone + dexmedetomidine) combined with preoperative and postoperative enteral nutrition support]; CG, control group [standard analgesia (sufentanil + dexamethasone + dexmedetomidine) combined with postoperative only enteral nutrition support]. *P < 0.05.

As shown in Figure 3, the times to first exhaust, first defecation, and postoperative hospitalization were all significantly shorter in the SG compared with the CG (*P* < 0.05).

### Immune indicators in both groups

**Figure 4:**
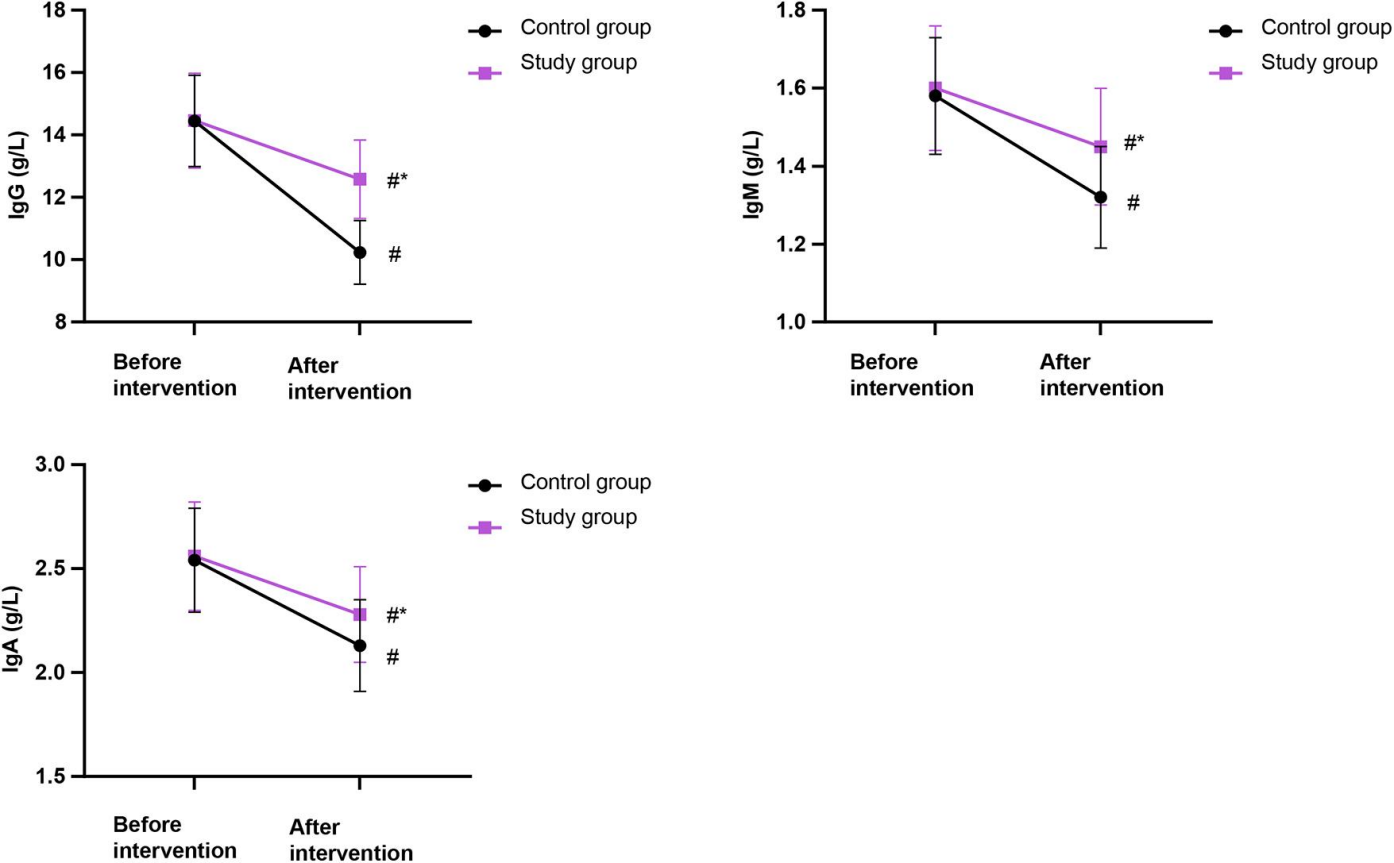
Comparison of immune indicators in both groups. IgG, immunoglobulin G; IgM, immunoglobulin M; IgA, immunoglobulin A (g/L). Data were presented as mean ± standard deviation. SG, study group [esketamine-containing analgesia (esketamine + sufentanil + dexamethasone + dexmedetomidine) combined with preoperative and postoperative enteral nutrition support]; CG, control group [standard analgesia (sufentanil + dexamethasone + dexmedetomidine) combined with postoperative only enteral nutrition support]. ^#^*P* < 0.05, compared with before intervention, *P < 0.05, compared with CG.

Figure 4 shows no significant difference in immune indicators (IgG, IgM, IgA) between the two groups before the intervention (*P* > 0.05). After the intervention, although the levels of these immunoglobulins decreased in both groups, they remained significantly higher in the SG than those in the CG (*P* < 0.05).

### Nutritional status in both groups

**Figure 5:**
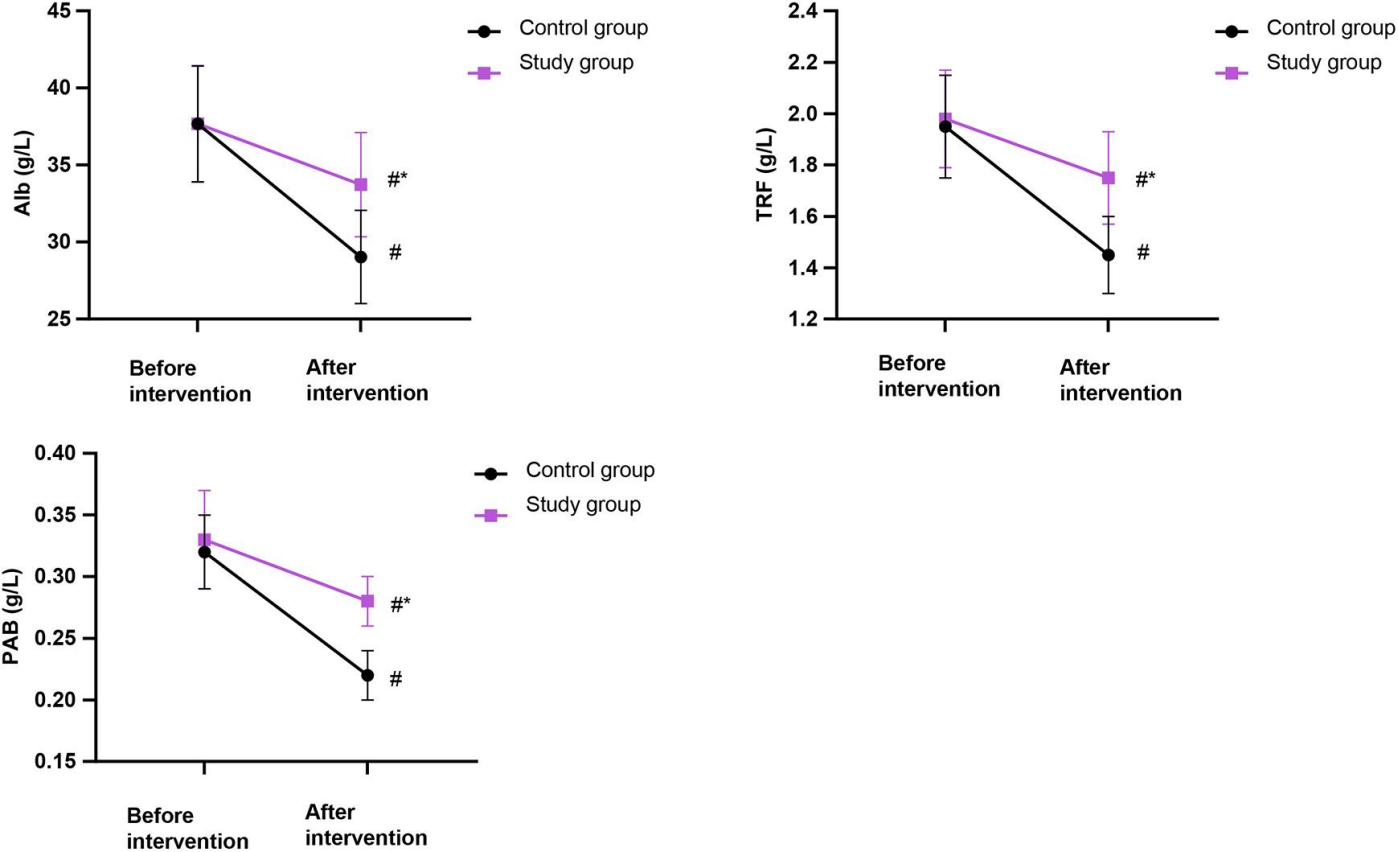
Comparison of nutritional status in both groups. Alb, albumin; TRF, transferrin; PAB, prealbumin (g/L). Data were presented as mean ± standard deviation. SG, study group [esketamine-containing analgesia (esketamine + sufentanil + dexamethasone + dexmedetomidine) combined with preoperative and postoperative enteral nutrition support]; CG, control group [standard analgesia (sufentanil + dexamethasone + dexmedetomidine) combined with postoperative only enteral nutrition support]. ^#^*P* < 0.05, compared with before intervention, *P < 0.05, compared with CG.

Figure 5 shows no significant differences in nutritional indexes (Alb, TRF, PAB) between the two groups before the intervention (*P* > 0.05). After the intervention, although levels of these markers decreased in both groups, the SG maintained significantly higher levels than the CG (*P* < 0.05).

### Quality of life in both groups

**Figure 6:**
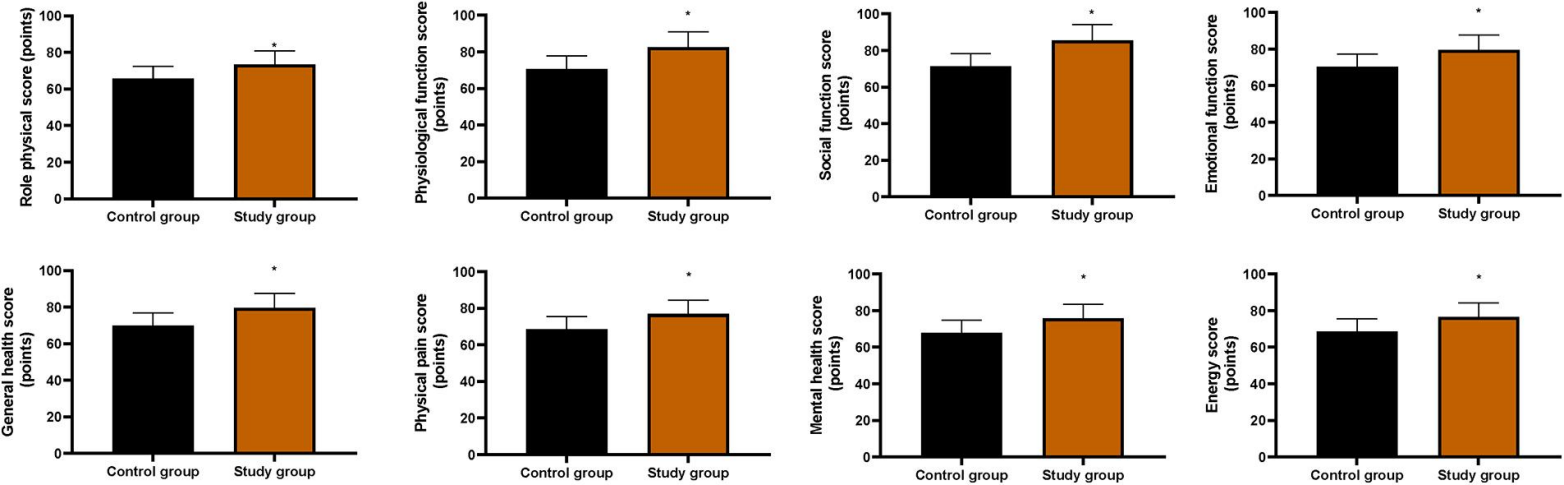
Quality of life in both groups. *P < 0.05. Scores of the eight domains of the 36-Item Short Form Health Survey (SF-36) were shown. Data were presented as mean ± standard deviation. SG, study group [esketamine-containing analgesia (esketamine + sufentanil + dexamethasone + dexmedetomidine) combined with preoperative and postoperative enteral nutrition support]; CG, control group [standard analgesia (sufentanil + dexamethasone + dexmedetomidine) combined with postoperative only enteral nutrition support]. *P < 0.05.

As shown in Figure 6, the SG showed significantly higher scores across all dimensions of the SF-36 compared with the CG (*P* < 0.05).

## Discussion

The incidence of lung cancer increases with age (14). Patients undergoing thoracoscopic radical resection for lung cancer often experience moderate to severe postoperative pain (15). With the growing adoption of enhanced recovery after surgery (ERAS) protocols, multimodal analgesia has been increasingly implemented for these patients (16). Effective analgesia can reduce the incidence of postoperative complications, such as respiratory depression (17). Although opioid-based intravenous analgesia remains the most common clinical approach, excessive opioid use can lead to adverse effects including respiratory depression, nausea, vomiting, and dizziness (18).

As a classic NMDA receptor antagonist, esketamine effectively reduces perioperative pain sensitization and enhances the analgesic effect of opioids, thereby reducing their required dosage (19). At the same time, esketamine can stimulate the central sympathetic nerve, release nerve catecholamines, and avoid hypotension, which is especially suitable for elderly patients with unstable hemodynamics (20). Recent thoracic surgery-specific evidence strengthens our findings. A study by Zhang et al. (21) showed that adding 0.2 mg kg^−^¹ esketamine to intravenous patient-controlled analgesia reduced 48 h sufentanil consumption by 35% after thoracoscopic lung resection. Yan et al. (22) achieved complete intraoperative opioid avoidance with systemic plus epidural esketamine and observed a lower incidence of chronic postsurgical pain at 3 months. In spontaneous ventilation video-assisted thoracic surgery, Fan et al. (23) recorded a 42% remifentanil sparing and better oxygenation, while Zeng et al. (24) demonstrated that a single intraoperative bolus halved the incidence of severe rebound pain after thoracic paravertebral block.

In this study, the SG demonstrated significantly lower VAS scores (at rest and during cough) and higher Ramsay sedation scores at 6, 12, 24, and 48 h postoperatively compared with the CG. The SG also required fewer analgesic pump compressions and a lower total analgesic dosage within 48 h, with a concurrent reduction in adverse reactions. These findings indicate that esketamine may effectively reduce the postoperative pain and agitation of patients, reduce the dosage of opioid analgesics, and reduce the occurrence of respiratory depression, hypotension, and other related adverse reactions caused by opioids, which is consistent with a previous study (25).

Studies have shown that early postoperative enteral nutrition provides essential energy, protein, fat, and carbohydrates, thereby helping to maintain postoperative metabolic balance and promote the recovery of intestinal digestion, absorption, and barrier function. It is therefore the preferred method of nutritional support (26). However, the rapid administration of a large volume of enteral nutrition shortly after surgery may lead to gastrointestinal intolerance, resulting in symptoms such as abdominal distension, pain, diarrhea, and gastric retention (27). Gastrointestinal intolerance compromises the effectiveness of enteral nutrition and increases the risk of treatment failure (28). Therefore, attention should be paid to the problem of postoperative enteral nutrition support intolerance, and practical measures should be taken to improve the compliance of postoperative enteral nutrition.

In our study, the outcomes displayed that IgG, IgM, IgA, Alb, TRF, and PAB levels were higher in the SG compared with the CG, suggesting that preoperative enteral nutrition support may enhance the immune function and nutritional status of patients receiving thoracoscopic radical resection of lung cancer. The underlying mechanisms for these improvements are likely multifactorial. We speculate that (1) the enteral nutrient solution contained various amino acids, proteins, fats, vitamins, organophosphates, trace elements, and other components, which were taken orally in a small dose before the operation and gradually increased. After absorption through the digestive tract, the nutritional status could be directly improved (29). These immune and nutritional benefits are supported by recent intervention studies. In a 2022 phase II trial of non-small cell lung cancer (NSCLC) patients, perioperative immunonutrition elevated serum IgG, IgA, IgM, and CD4+ counts while increasing pre-Alb levels (30). A 2023 meta-analysis pooling 28 randomized controlled trials (RCTs) confirmed significant standardized mean differences for IgG (0.98), IgM (1.15), and prealbumin (0.73) when immunonutrition was administered ≥7 days before surgery (31). More importantly, (2) the preoperative administration of a small dose of enteral nutrient solution might have primed the gastrointestinal tract, enhancing its tolerance to postoperative feeding. This could help maintain gut barrier function, reduce postoperative intolerance, and thereby improve the overall success of nutritional support, which is crucial for modulating immune and metabolic responses after surgery (32). Protection of the gut barrier has been documented by Li and Wang, who observed decreased intestinal permeability markers (DAO, D-lactate) and fewer episodes of bacterial translocation when low-dose EN was commenced 5 days pre-operatively (33). However, these mechanisms remain speculative within the context of our study, and further investigation is warranted to elucidate the precise pathways.

Furthermore, our study found that compared with the CG, the SG showed significantly shorter times to first exhaust, first defecation, and postoperative hospitalization, along with higher scores across all SF-36 dimensions. These results suggest that the combination of preoperative nutritional support and esketamine is associated with accelerated postoperative recovery and improved quality of life in elderly patients undergoing thoracoscopic lung cancer surgery, which is consistent with previous studies (34, 35).

This study has several limitations that must be emphasized when interpreting its findings. Firstly and most importantly, the non-randomized, retrospective design is a fundamental limitation. The allocation of patients to the study and control groups was based on clinical practice rather than randomization, which inherently carries a risk of selection bias and unmeasured confounding. Although baseline characteristics were comparable, this design significantly weakens the strength of causal inference regarding the effects of the combined intervention. Secondly, the single-center nature of the study limits the generalizability of our results. The findings may be influenced by specific institutional protocols, surgical expertise, and patient populations, and thus may not be directly applicable to other settings. Thirdly, the absence of blinding is a notable source of potential bias. The patients and clinicians involved in outcome assessment were aware of the group assignments, which could have influenced the reporting of subjective outcomes such as pain scores (VAS) and quality of life (SF-36). Finally, the follow-up period was relatively short, focused exclusively on the immediate postoperative hospitalization phase. This design does not allow for any conclusions regarding long-term outcomes, including 30-day readmission or complication rates, overall survival, or the sustainability of the observed improvements in immune function and quality of life. Future large-scale, multicenter, prospective, randomized, double-blind trials with long-term follow-up are essential to confirm our findings and establish the long-term benefits of this combined approach.

## Conclusion

Our findings suggest that for elderly patients after thoracoscopic radical resection of lung cancer, the combined approach of preoperative nutritional support and esketamine may lead to improved analgesia, enhanced recovery, better preservation of immune and nutritional function, and an improved quality of life, suggesting it may be a beneficial strategy within an enhanced recovery protocol.

## Data Availability

The raw data supporting the conclusions of this article will be made available by the authors, without undue reservation.

## References

[1] The Lancet. Lung cancer: some progress, but still a lot more to do. Lancet. (2019) 394:1880. 10.1016/S0140-6736(19)32795-3 31777378

[2] Brunelli A Charloux A Bolliger CT Rocco G Sculier JP Varela G ERS/ESTS clinical guidelines on fitness for radical therapy in lung cancer patients (surgery and chemo-radiotherapy). Eur Respir J. (2009) 34:17–41. 10.1183/09031936.00184308 19567600

[3] Lowczak A Kolasinska-Cwikla A Ćwikła JB Osowiecka K Palucki J Rzepko R Outcomes of patients with clinical stage I–IIIA large-cell neuroendocrine lung cancer treated with resection. J Clin Med. (2020) 9:1370. 10.3390/jcm9051370 32392725 PMC7290504

[4] Chai T Lin Y Kang M Lin J. Thoracotomy versus video-assisted thoracoscopic resection of lung cancer: a protocol for a systematic review and meta-analysis. Medicine. (2019) 98:e14646. 10.1097/MD.0000000000014646 30855453 PMC6417539

[5] Hey JC. Lung cancer in elderly patients. Clin Geriatr Med. (2003) 19:139–55. 10.1016/S0749-0690(02)00067-8 12735119

[6] Huyan T Hu X Peng H Zhu Z Li Q Zhang W. Perioperative dexmedetomidine reduces delirium in elderly patients after lung cancer surgery. Psychiatr Danub. (2019) 31:95–101. 10.24869/psyd.2019.95 30948695

[7] Mercadante S Arcuri E Santoni A. Opioid-induced tolerance and hyperalgesia. CNS Drugs. (2019) 33:943–55. 10.1007/s40263-019-00660-0 31578704

[8] Oldham JM. Opioids. J Psychiatr Pract. (2020) 26:1–2. 10.1097/PRA.0000000000000444 31913964

[9] Wick EC Grant MC Wu CL. Postoperative multimodal analgesia pain management with nonopioid analgesics and techniques: a review. JAMA Surg. (2017) 152:691–7. 10.1001/jamasurg.2017.0898 28564673

[10] Qiu D Wang XM Yang JJ Chen S Yue CB Hashimoto K Effect of intraoperative esketamine infusion on postoperative sleep disturbance after gynecological laparoscopy: a randomized clinical trial. JAMA Netw Open. (2022) 5:e2244514. 10.1001/jamanetworkopen.2022.44514 36454569 PMC9716381

[11] Zhang X Edwards BJ. Malnutrition in older adults with cancer. Curr Oncol Rep. (2019) 21:80. 10.1007/s11912-019-0829-8 31359189

[12] Yan X Zhou FX Lan T Xu H Yang XX Xie CH Optimal postoperative nutrition support for patients with gastrointestinal malignancy: a systematic review and meta-analysis. Clin Nutr. (2017) 36:710–21. 10.1016/j.clnu.2016.06.011 27452745

[13] Deftereos I Kiss N Isenring E Carter VM Yeung JM. A systematic review of the effect of preoperative nutrition support on nutritional status and treatment outcomes in upper gastrointestinal cancer resection. Eur J Surg Oncol. (2020) 46:1423–34. 10.1016/j.ejso.2020.04.008 32336624

[14] Gajra A Akbar SA Din NU. Management of lung cancer in the elderly. Clin Geriatr Med. (2016) 32:81–95. 10.1016/j.cger.2015.08.008 26614862

[15] Liu X An J. Effects of serratus anterior plane block and thoracic paravertebral nerve block on analgesia, immune function and serum tumor markers in patients after thoracoscopic radical resection of lung cancer. Nagoya J Med Sci. (2022) 84:506–15. 10.18999/nagjms.84.3.506 36237885 PMC9529616

[16] Harky A Clarke CG Kar A Bashir M. Epidural analgesia vs. paravertebral block in video-assisted thoracoscopic surgery. Interact Cardiovasc Thorac Surg. (2019) 28:404–6. 10.1093/icvts/ivy265 30169855

[17] Soffin EM Memtsoudis SG. Anesthesia and analgesia for total knee arthroplasty. Minerva Anestesiol. (2018) 84:1406–12. 10.23736/S0375-9393.18.12383-2 29808972

[18] Chang CY Tu YK Kao MC Shih PC Su IM Lin HY Effects of opioids administered via intravenous or epidural patient-controlled analgesia after caesarean section: a network meta-analysis of randomised controlled trials. EClinicalMedicine. (2023) 56:101787. 10.1016/j.eclinm.2022.101787 36590790 PMC9800204

[19] Witkin JM Martin AE Golani LK Xu NZ Smith JL. Rapid-acting antidepressants. Adv Pharmacol. (2019) 86:47–96. 10.1016/bs.apha.2019.03.002 31378256

[20] Zhang Y Cui F Ma JH Wang DX. Mini-dose esketamine-dexmedetomidine combination to supplement analgesia for patients after scoliosis correction surgery: a double-blind randomised trial. Br J Anaesth. (2023) 131:385–96. 10.1016/j.bja.2023.05.001 37302963

[21] Zhang A Zhou Y Zheng X Zhou W Gu Y Jiang Z Effects of S-ketamine added to patient-controlled analgesia on early postoperative pain and recovery in patients undergoing thoracoscopic lung surgery: a randomized double-blinded controlled trial. J Clin Anesth. (2024) 92:111299. 10.1016/j.jclinane.2023.111299 37939610

[22] Yan H Chen W Chen Y Gao H Fan Y Feng M Opioid-free vs. opioid-based anesthesia on postoperative pain after thoracoscopic surgery: the use of intravenous and epidural esketamine. Anesth Analg. (2023) 137:399–408. 10.1213/ANE.0000000000006547 37267129

[23] Fan Q Luo J Zhou Q Zhang Y Zhang X Li J Esketamine opioid-free intravenous anesthesia versus opioid intravenous anesthesia in spontaneous ventilation video-assisted thoracic surgery: a randomized controlled trial. Front Oncol. (2023) 13:1145953. 10.3389/fonc.2023.1145953 37324000 PMC10266098

[24] Zeng X Zhang X Jiang W Zhou X. Efficacy of intravenous administration of esketamine in preventing and treating rebound pain after thoracic paravertebral nerve block: a prospective randomized, double-blind, placebo-controlled trial. Drug Des Devel Ther. (2024) 18:463–73. 10.2147/DDDT.S448336 38384750 PMC10880457

[25] Lei Y Liu H Xia F Gan S Wang Y Huo W Effects of esketamine on acute and chronic pain after thoracoscopy pulmonary surgery under general anesthesia: a multicenter-prospective, randomized, double-blind, and controlled trial. Front Med. (2021) 8:693594. 10.3389/fmed.2021.693594 PMC8455819 34568362

[26] Wobith M Weimann A. Oral nutritional supplements and enteral nutrition in patients with gastrointestinal surgery. Nutrients. (2021) 13:2655. 10.3390/nu13082655 34444812 PMC8400187

[27] Zhang W Zhu NN Jiang HJ Tao XB Lu WH Shen HC Prevention of underfeeding during enteral nutrition after gastrectomy in adult patients with gastric cancer: an evidence utilization project. JBI Evid Implement. (2020) 19:198–207. 10.1097/XEB.0000000000000248 32815858 PMC8183477

[28] Wang Y Li Y Li Y Li H Zhang D. Enteral feeding strategies in patients with acute gastrointestinal injury: from limited to progressive to open feeding. Nutrition. (2024) 117:112255. 10.1016/j.nut.2023.112255 37897987

[29] Martin K Gardner G. Home enteral nutrition: updates, trends, and challenges. Nutr Clin Pract. (2017) 32:712–21. 10.1177/0884533617701401 28437132

[30] Akcam TI Tekneci AK Kavurmaci O Ozdil A Ergonul AG Turhan K The significance of immunonutrition nutritional support in patients undergoing postoperative adjuvant chemotherapy for lung cancer: case–control study. World J Surg Oncol. (2023) 21:183. 10.1186/s12957-023-03073-y 37337249 PMC10280930

[31] Cui J Liu Y Li F Zhuo W Huang L Xu C Evidence-based guideline on immunonutrition in patients with cancer. Precis Nutr. (2023) 2:e00031. 10.1097/PN9.0000000000000031

[32] Kaya SO Akcam TI Ceylan KC Samancılar O Ozturk O Usluer O. Is preoperative protein-rich nutrition effective on postoperative outcome in non-small cell lung cancer surgery? A prospective randomized study. J Cardiothorac Surg. (2016) 11:14. 10.1186/s13019-016-0407-1 26782276 PMC4717613

[33] Li Q Wang J. The application and mechanism analysis of enteral nutrition in clinical management of chronic diseases. Nutrients. (2025) 17:450. 10.3390/nu17030450 39940308 PMC11820659

[34] Martin RC 2nd Agle S Schlegel M Hayat T Scoggins CR Mcmasters KM Efficacy of preoperative immunonutrition in locally advanced pancreatic cancer undergoing irreversible electroporation (IRE). Eur J Surg Oncol. (2017) 43:772–9. 10.1016/j.ejso.2017.01.002 28162818

[35] Min M Du C Chen X Xin W. Effect of subanesthetic dose of esketamine on postoperative rehabilitation in elderly patients undergoing hip arthroplasty. J Orthop Surg Res. (2023) 18:268. 10.1186/s13018-023-03728-2 37009879 PMC10069053

